# Knockdown of Glycolysis-Related LINC01070 Inhibits the Progression of Breast Cancer

**DOI:** 10.7759/cureus.60093

**Published:** 2024-05-11

**Authors:** Qiang Hu, Yiduo Wang, Weipu Mao

**Affiliations:** 1 Urology, Zhongda Hospital, Southeast University, Nanjing, CHN

**Keywords:** nomogram, risk model, linc01070, lncrnas, glycolysis, breast cancer

## Abstract

Accumulative evidence confirms that glycolysis and long non-coding RNAs (lncRNAs) are closely associated with tumor development. The aim of this study was to construct a novel prognostic model based on glycolysis-related lncRNAs (GRLs) in breast cancer patients. By performing Pearson correlation analysis and Lasso regression analysis on differentially expressed genes and lncRNAs associated with glycolysis in the Cancer Genome Atlas (TCGA) and Gene Set Enrichment Analysis (GSEA) datasets, we identified nine GRLs and constructed associated prognostic risk signature. Kaplan-Meier survival analysis and univariate and multivariate Cox analysis showed that patients in the low-risk group had a better prognosis. The receiver operator characteristics (ROC) curves showed that the area under the curve (AUC) of the prognostic risk signature predicting patients' overall survival at 1-, 3- and 5- years was 0.78, 0.71, and 0.71, respectively. Moreover, the validation curves also showed that the signature had better diagnostic efficacy and clinical predictive power. Furthermore, clone formation assay, EdU assay, and Transwell assay showed that knockdown of LINC01070 inhibited breast cancer progression. We developed a prognostic risk-associated GRLs signature that can accurately predict the breast cancer patient's prognostic status, and LINC01070 can be used as a potential biomarker for the prognosis of breast cancer patients.

## Introduction

Breast cancer is one of the most common malignancies, and breast cancer in women has surpassed lung cancer as the type of malignancy that causes the highest mortality rate in women. According to statistics, there will be approximately 2.26 million new cases of breast cancer worldwide in 2020, accounting for 11.7% of all new cancer cases [1, 2]. Breast cancer is highly prevalent, aggressive, and highly susceptible to metastasis and recurrence, with approximately one-third of female patients experiencing death despite radical mastectomy [3]. Given the genetic heterogeneity among individuals with breast tumors, this results in extremely challenging breast cancer treatment [4]. Therefore, it is particularly important to explore, discover, and utilize effective molecular markers for the early diagnosis of tumors [5].

Long non-coding RNAs (lncRNAs) are a class of endogenous RNA molecules with a nucleotide length greater than 200 bp [6]. They are found in the nucleus or cytoplasm of cells and have a relatively conserved secondary structure that allows them to regulate gene expression by interacting with DNA molecules, RNA molecules, and proteins [7]. LncRNAs function in cells in three main ways: through chromatin remodeling to mediate the silencing of certain genes; through RNA stabilization and transcriptional regulation; and through post-transcriptional regulation of genes [8]. LncRNAs are involved in various aspects of cellular homeostasis, such as apoptosis, migration, proliferation, gene transcription, and post-transcriptional processing [9]. Many studies have demonstrated that lncRNAs play a regulatory role in the progression of many cancers, including breast cancer [10].

Unlike normal cells, breast cancer cells are accompanied by unlimited cell proliferation, accelerated growth, and abnormal energy metabolism [11]. One of the main features of abnormal energy metabolism is glucose metabolism. Glycolysis is a well-recognized feature of energy metabolism in tumor cells and is known as the Warburg effect [12, 13]. The Warburg effect is the preferred mode of energy metabolism in cancer cells and is necessary for tumor growth, which is facilitated by aerobic glycolysis to produce lactic acid, which provides energy to cancer cells. Even in the presence of sufficient oxygen, most tumor cells generate energy through glycolysis, increasing glucose uptake and glycolytic activity to produce large amounts of lactate [14]. This metabolic approach allows for the rapid synthesis of adenosine triphosphate (ATP), which provides a selective survival advantage for cancer cells [15]. Therefore, the glycolytic pathway has received much attention as a molecular target for tumor therapy. The purpose of our study was to construct a novel prognostic model based on glycolysis-related lncRNAs (GRLs) in breast cancer patients to develop promising prognostic indicators and possible therapeutic targets for breast cancer patients.

## Materials and methods

Data download and extraction

Count and transcripts per million (TPM) data of The Cancer Genome Atlas (TCGA)-Breast Cancer transcriptome were downloaded from the Genomic Data Commons (GDC) database, and whole transcriptome data of 1,113 breast cancer patients and 113 normal patients were obtained. Differential expression analysis was performed using count data, TPM data were log2(TPM+1) processed, and correlation and regression analyses were performed. In the Gene Set Enrichment Analysis (GSEA) database, we screened nine glycolysis-related pathways and the number of related genes (Table 1).

**Table 1:** Glycolysis-related terms. Abbreviations: HIF-1α, hypoxia-inducible factor-1α; PPARG, peroxisome proliferator-activated receptor gamma; KEGG, Kyoto encyclopedia of genes and genomes.

Terms	Gene size
Reactome glycolysis	72
Reactome regulation of glycolysis by fructose 2 6 bisphosphate metabolism	12
HIF-1α and PPARG regulation of glycolysis	8
Aerobic glycolysis	12
Glycolysis and gluconeogenesis	45
Glycolysis in senescence	11
Hallmark glycolysis	200
Mootha glycolysis	21
KEGG glycolysis gluconeogenesis	62

Using the DESeq2 package, differentially expressed lncRNAs (DELs) and differentially expressed genes (DEGs) were obtained according to the screening criteria of logFC >= 2 and adjusted P-value < 0.05. Pearson correlation analysis screened out glycolysis-related genes with an absolute value of correlation > 0.6 and P-value < 0.05 with differential GRLs.

Identification of differential GRLs

The clinicopathological data of all patients were downloaded from the TCGA database, and samples with no expression profile data, duplicate samples, and data with follow-up time ≤ 10 days were removed, resulting in a total of 1,011 clinical samples with complete survival time and survival status (Table 2).

**Table 2:** Clinical characteristics of selected patients with breast cancer in TCGA database. Abbreviations: PR, progesterone receptor; ER, estrogen receptor; HER2, human epithelial growth factor receptor 2; TCGA, The Cancer Genome Atlas. Note: The data has been represented as N (%).

Characteristic	Group	Size (%)
Age (years)	<= 50	312 (30.86)
	> 50	699 (69.14)
PR	Positive	654 (64.69)
	Negative	316 (31.26)
	Indeterminate/Unknow	41 (4.05)
ER	Positive	755 (74.68)
	Negative	217 (21.46)
	Indeterminate/Unknow	39 (3.86)
HER2	Positive	141 (13.95)
	Negative	518 (51.24)
	Indeterminate/Unknow	352 (34.82)
Stage	Stage I	200 (19.78)
	Stage II	562 (55.59)
	Stage III	229 (22.65)
	Stage IV	20 (1.98)
T stage	T1	272 (26.90)
	T2	570 (56.38)
	T3	130 (12.86)
	T4	39 (3.86)
N stage	N0	468 (46.29)
	N1	344 (34.03)
	NX	199 (19.68)
M stage	M0	832 (82.29)
	M1	22 (2.18)
	MX	157 (15.53)
Status	Alive	909 (89.91)
	Dead	102 (10.09)

The 1,011 patients were randomly divided into a training group (607 patients) and a validation group (404 patients) according to the ratio of 6:4. The lncRNA expression data were combined with survival data, differential GRLs were screened by lasso cox regression using the glmnet package. Prognostic risk models were constructed based on the expression levels of the screened lncRNAs, clinical samples were divided into low-risk group and high-risk group based on the calculated risk scores, and prognostic model groupings and survival status were visualized in the training and validation groups.

Assessing the impact of risk models on overall survival (OS)

Kaplan-Meier survival curves were constructed using survminer and survival packages in the training and validation groups, and log-rank test analysis was performed to assess the effect of the risk model on OS. Univariate Cox proportional risk regression analysis was performed based on the constructed model and known clinical information (age, tumor-lymph node-metastasis (TNM)-stage, human epithelial growth factor receptor 2 (HER2), estrogen receptor (ER), and progesterone receptor (PR)). Variables with P-value < 0.1 in univariate analysis were included in multivariate Cox analysis to detect prognosis-related independent risk factors.

Construction and validation of OS nomogram

Based on the results of the multivariate Cox regression analysis in the previous step, a nomogram for OS was constructed using the rms package and the survivor package, and the receiver operating characteristic (ROC) curves and calibration curves were used to assess the predictive effect of the nomogram for 1-year, 3-year and 5-year OS.

Screening for prognosis-related differential GLRs

In the whole cohort, Kaplan-Meier survival curves were constructed using survminer and survival packages, and log-rank test analysis was performed to assess the effect of differential GLRs on OS of breast cancer patients, and differential GLRs associated with prognosis were screened.

Construction of lncRNA-microRNA-mRNA network

Prognosis-related differential GLRs-related miRNAs and glycolytic genes were predicted using the Starbase database (https://starbase.sysu.edu.cn/), and lncRNA-microRNA-mRNA network maps were constructed in the ggalluvial package.

GSEA analysis

GSEA analysis using the clusterProfiler package identified different functional phenotypes between the high-risk and low-risk groups, and the mRNA expression profiles of patients were assessed using the Kyoto Encyclopedia of Genes and Genomes (KEGG). P<0.05 for the Reactome pathway was considered significantly different.

Cell culture and transfection

Human breast cancer cell line BT-549 (Cat: TCHu 93) was purchased from the Cell Bank of the Chinese Academy of Sciences (Shanghai, China). RPMI-1640 medium (Gibco; Thermo Fisher Scientific, USA) supplemented with 10% fetal bovine serum (FBS, Gibco; Thermo Fisher Scientific, USA) and 1% penicillin/streptomycin (Gibco; Thermo Fisher Scientific, USA) was used to maintain cell line. The cell lines were stored at -80°C using the CELLSAVING reagent (NCM, Suzhou, China). The small interfering RNAs specifically targeting LINC01070 (si-LINC01070) were purchased from IBSBIO (Shanghai, China). Transient transfection was performed at 30%-50% cell confluence using Lipofectamine 3000 (Thermo Fisher Scientific, USA). The sequence of si-LINC01070 is AGAAUUGUGAGGCUUUAUCUU.

Colony formation assay

The treated cells were inoculated in 6-well plates at 500 cells per well and cultured for 2 weeks. After 2 weeks of incubation, the medium was discarded and the cell colonies were washed three times with phosphate buffer solution (PBS), fixed with 75% ethanol, and stained with 0.1% crystal violet (Vicmed, China). The colonies were subsequently counted and photographed.

5-Ethynyl-2′-deoxyuridine (EdU) assay

Transfected cells were inoculated into 12-well plates (Corning, USA). Cells were fixed after incubation for 2 h using 10 μM EdU solution (Ruibo Bio, Guangzhou, China). Cells were stained according to the manufacturer's instructions, and finally, images were taken after staining the nuclei with Hoechst.

Transwell assay

The invasion assay was performed in the upper chamber pre-coated with 100 μL Matrigel (BD Biosciences, USA). Specifically, transfected cells were inoculated into the upper chamber, and then 500 μL of medium containing 10% FBS was added to the lower chamber. After 12-24 hours of incubation, the cells on the surface of the lower chamber were fixed with anhydrous ethanol, stained with 0.1% crystalline violet (Vicmed, China), and then photographed and counted.

Statistical analysis

GraphPad Prism 8.3 software (San Diego, USA) and R-Studio (Boston, USA) were used for statistical analyses in this study. P-values were considered statistically significant when calculated at <0.05.

## Results

Screening for differential GRGs and differential GRLs

**Figure 1:**
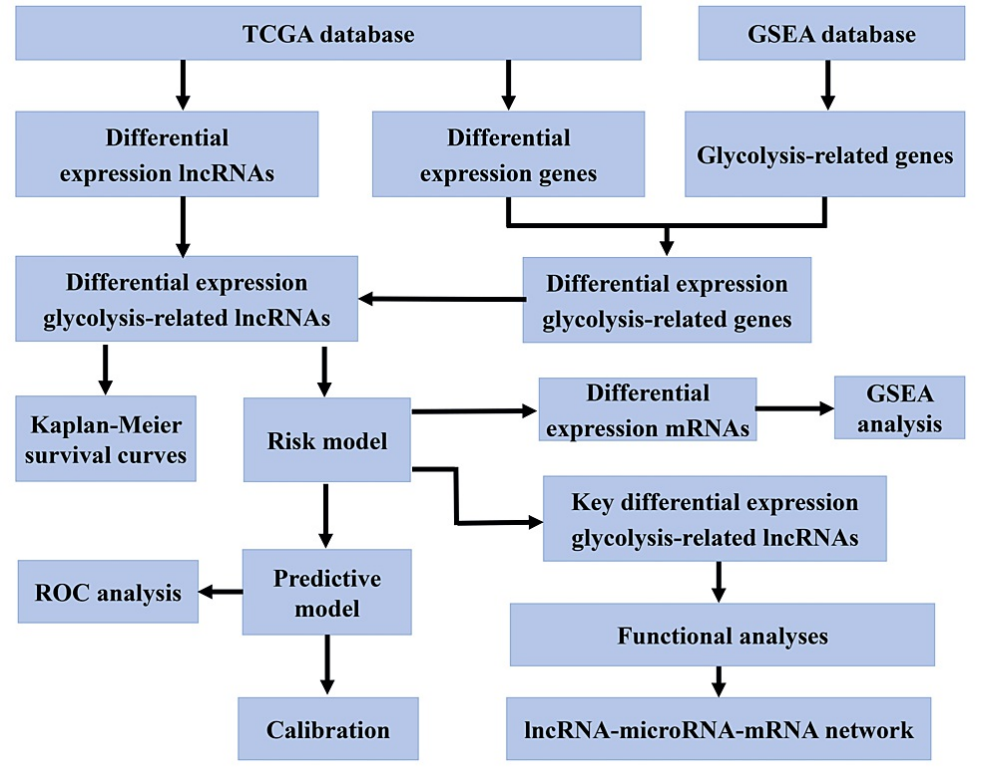
The workflow of this study. Abbreviations: TCGA, The Cancer Genome Atlas; GSEA: Gene Set Enrichment Analysis; lncRNA: long non-coding RNAs

The workflow of this study is shown in Figure 1.

**Figure 2:**
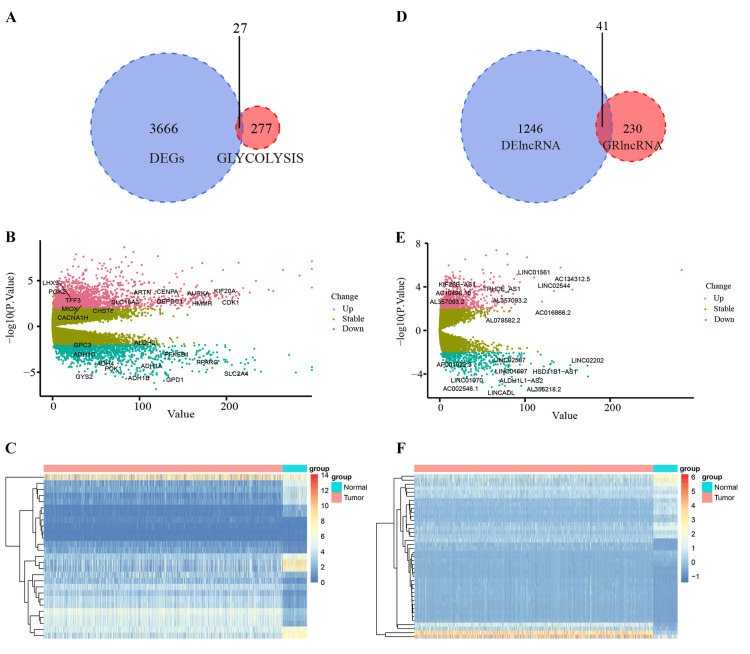
Differential GRGs and Differential GRLs were selected between breast cancers and normal tissues. A. 27 differential GRGs were detected between 3,693 DEGs and 304 GRGs. B, C. Volcano plot and heatmap of 27 differential GRGs were labeled. D. 41 differential GRLs were detected between 1,287 DELs and 271 GRLs. E, F. Volcano plot and heatmap of 41 differential GRLs were labeled. Abbreviations: DEG, differentially expressed lncRNA; GRL, glycolysis-related lncRNA; GRG, glycolysis-related genes

Based on the screening criteria of logFC>=2 and adjusted P-value <0.05, we obtained 3,694 DEGs and 1,287 DELs from 1,113 breast cancer patients and 113 normal patients in the TCGA database, and a total of 304 glycolysis-related genes (GRGs) in the GSEA database. By Pearson correlation analysis between different genes and lncRNAs, we finally screened 27 differential GRGs and 41 differential GRLs (Figure 2A, 2D), of which 15 and 20 were up-regulated differential GRGs and differential GRLs, respectively, 12 and 21 were down-regulated differential GRGs and differential GRLs, respectively (Figure 2B-2C and Figure 2E-2F).

Construction of the risk model

**Figure 3:**
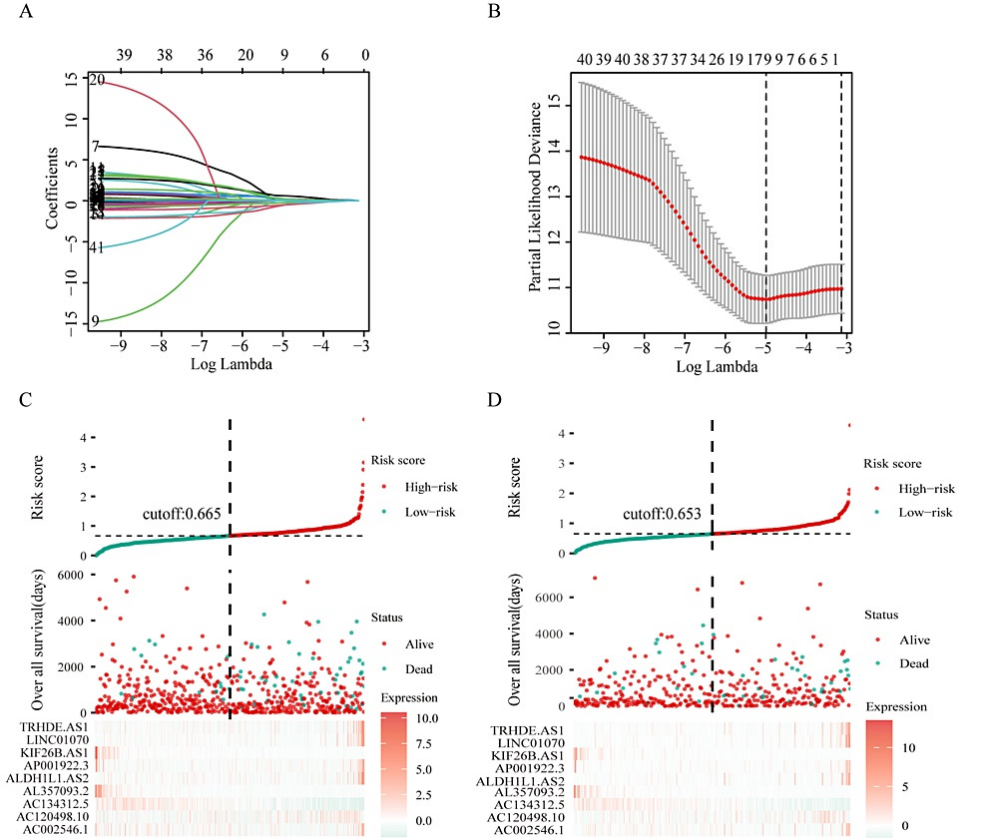
Construction of a risk signature consisting of nine differential GRLs. A. Lasso coefficient profiles of the 41 differential lncRNAs. B. Mean-squared error plot of the lowest point of the red curve. C, D. All patients were divided into high-risk and low-risk groups using the median risk score as the cut-off point in the training (C) and validation (D) groups. Abbreviations: GRL, glycolysis-related lncRNAs; lncRNA: long non-coding RNAs

Using lasso regression analysis, we identified nine differential GRLs (TRHDE.AS1, LINC01070, KIF26B.AS1, AP001922.3, ALDH1L1.AS2, AL357093.2, AC134312.5, AC120498.10, AC002546.1) associated with prognostic risk from the 41 differential GRLs (Figure 3A, 3B).

**Figure 4:**
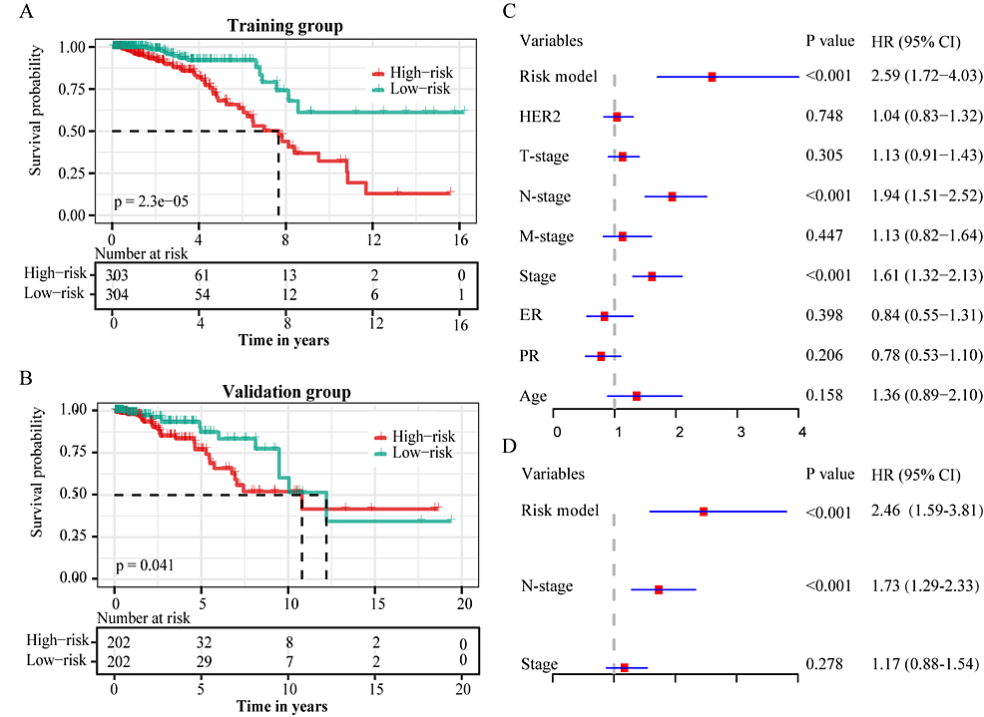
The risk score model and Cox proportional hazards regression model for predicting prognosis of breast cancer patients. A, B. Risk score model for predicting prognosis in the training (A) and validation (B) groups. C. Univariate cox proportional hazards regression model of variables for overall survival. D. Multivariate cox proportional hazards regression model of variables for overall survival.

A risk model was constructed based on the lasso regression analysis, and patients were divided into low-risk and high-risk groups in the training and validation groups. Heat maps showed significant differences in the expression profiles of nine prognosis-related GRLs in the two groups (Figure 3C, 3D). Subsequently, Kaplan-Meier survival curves showed that OS was significantly lower in high-risk patients than in low-risk patients in both the training (p<0.001) and validation (p=0.041) groups (Figure 4A, 4B).

In addition, univariate and multivariate Cox regression analyses revealed the risk model, N stage, and stage as independent risk factors for OS (Figure 4C, 4D).

Construction and validation of OS nomogram

**Figure 5:**
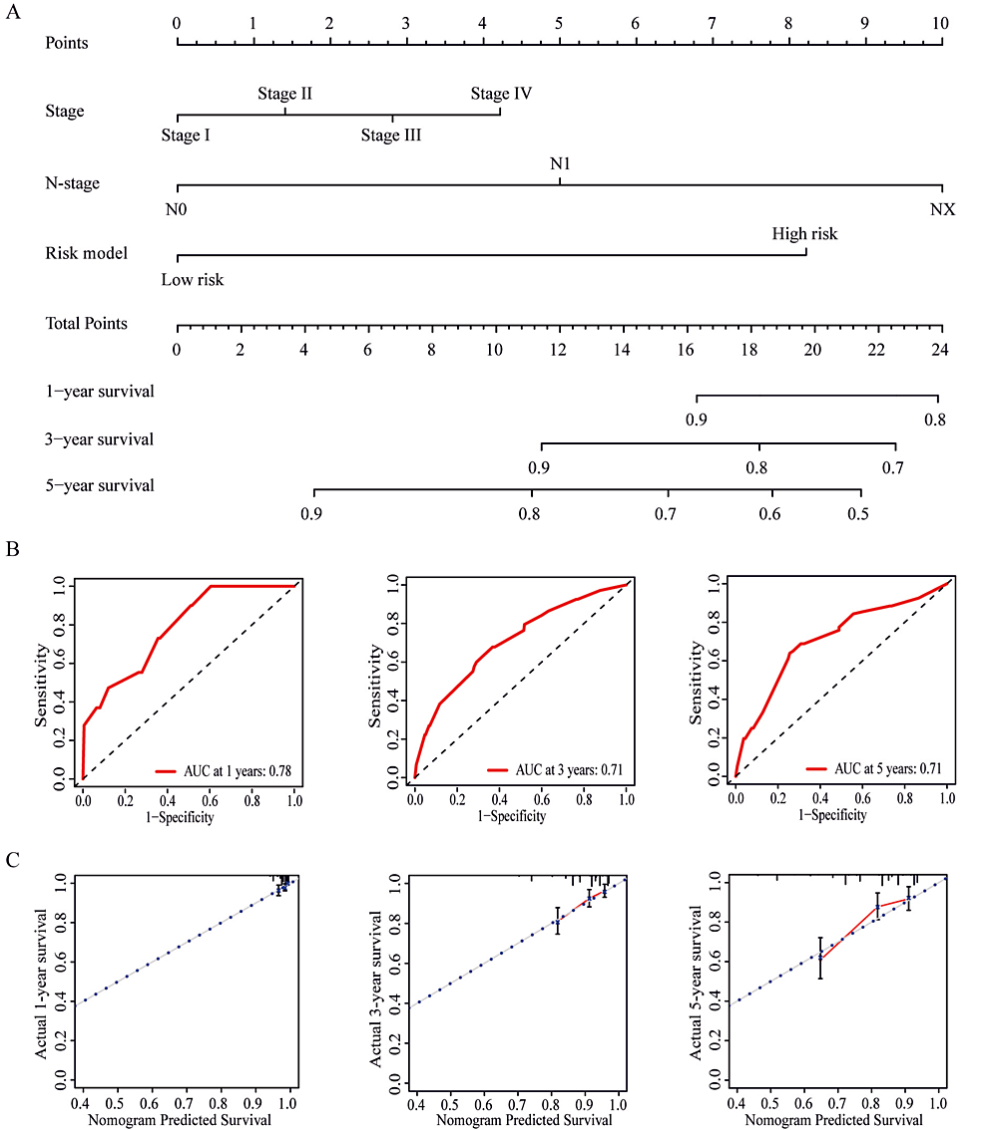
Construction of nomogram prediction models. A. Nomogram for predicting 1-year, 3-year and 5-year survival. B. Receiver operating characteristic (ROC) curves for survival at 1, 3 and 5 years. C. Calibration curves for predicting survival probabilities at 1, 3 and 5 years.

Based on the results of multivariate Cox regression analysis, we constructed an OS nomogram by combining the risk model, N stage, and stage (Figure 5A).

**Figure 6:**
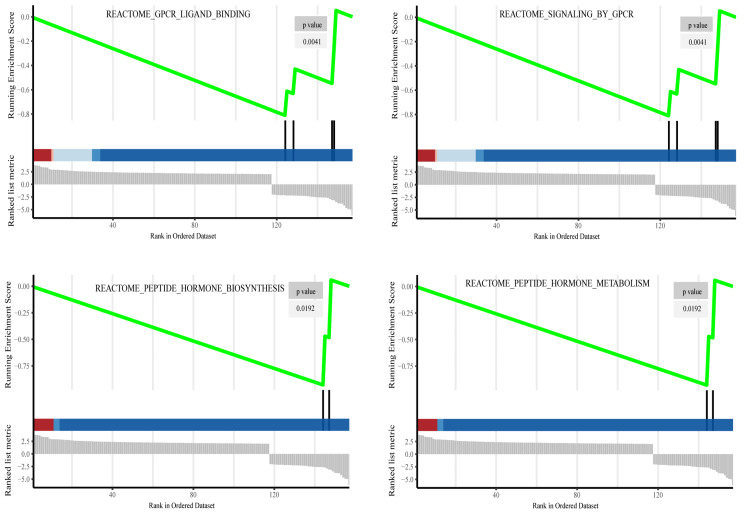
Functional enrichment analysis of the risk model by GSEA analysis. Abbreviation: GSEA, Gene Set Enrichment Analysis; GPCR, G-protein-coupled receptors

The ROC curve showed that the area under the curve (AUC) of the nomogram predicting 1-year, 3-year, and 5-year OS was 0.78, 0.71, and 0.71, respectively (Figure 5B). In addition, the calibration curve also showed that the prognostic nomogram predicted 1-year, 3-year, and 5-year survival rates with actual survival rates with high accuracy (Figure 5C). Moreover, GSEA analysis revealed that G-protein-coupled receptors (GPCR)ligand binding, signaling by GPCR, peptide hormone biosynthesis, and peptide hormone metabolism pathways may be involved in differences in glycolysis between the low-risk and high-risk groups (Figure 6).

Knockdown of LINC01070 inhibited breast cancer progression

**Figure 7:**
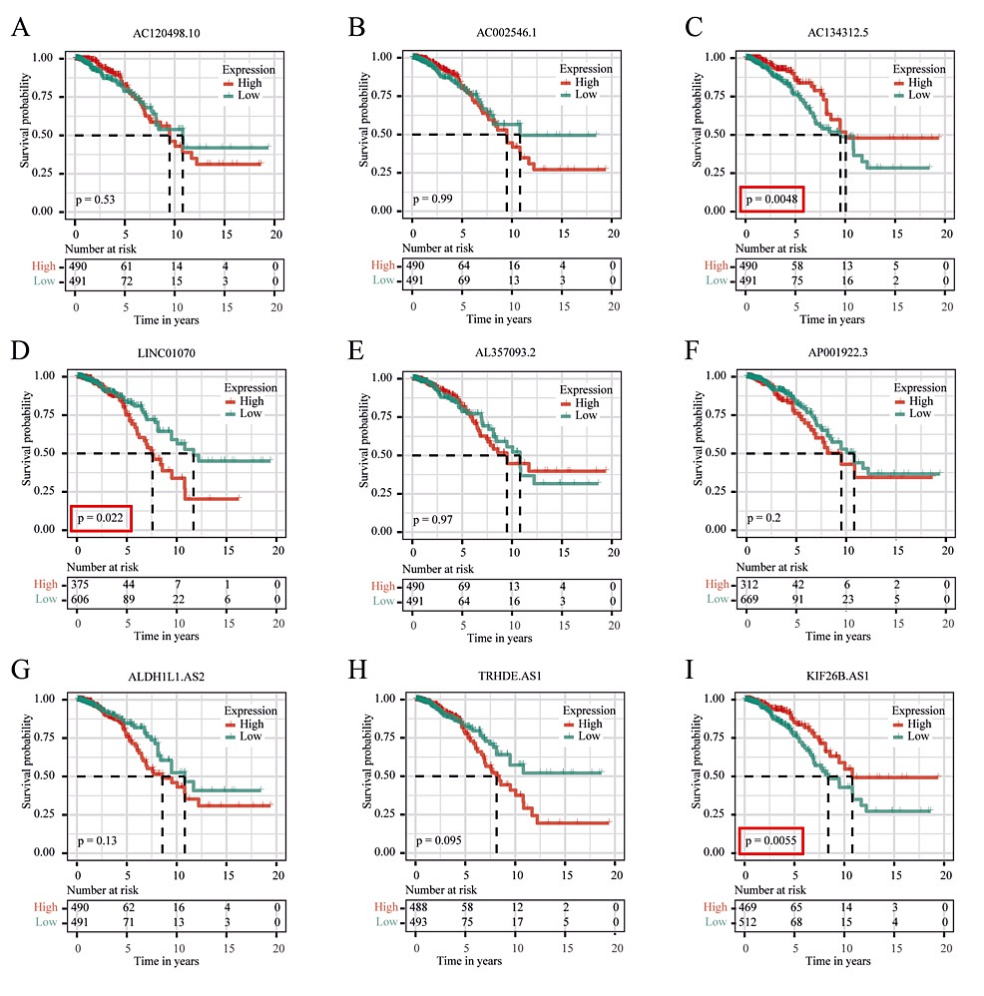
Kaplan-Meier survival curves for 9 differential GRLs in breast cancer patients. A. AC120498.10. B. AC002546.1. C. AC134312.5. D. LINC01070. E. AL357093.2. F. AP001922.3. G. ALDH1L1.AS2. H. TRHDE.AS1. I. KIF26B.AS1.

GRLs were classified into low and high-expression groups based on the median expression levels of breast cancer patients in the TCGA database. Kaplan-Meier survival curves showed that high LINC01070, high KIF26B.AS1, and low AC134312.5 were associated with poorer OS (p<0.05) (Figure 7).

**Figure 8:**
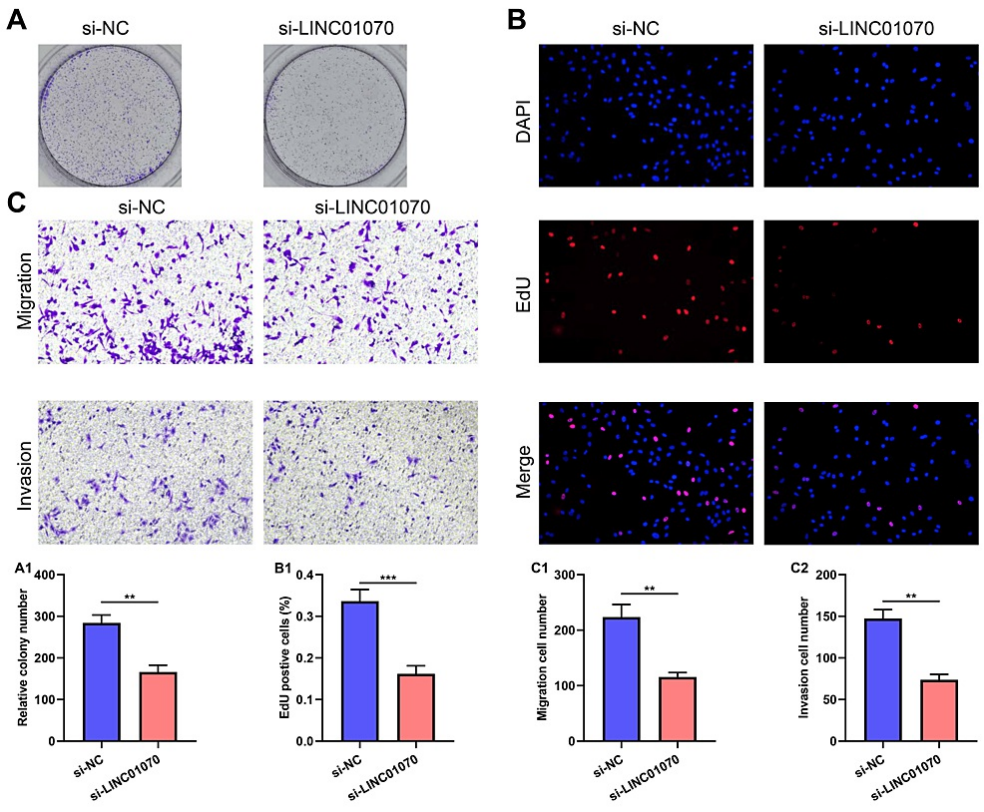
Knockdown of LINC01070 inhibited breast cancer progression. A. Growth ability of BT-549 cells (H) transfected with si-NC and si-LINC01070 assessed by clone formation assay. B. Growth ability of BT-549 cells (H) transfected with si-NC and si-LINC01070 assessed by EdU assay. C. Migration and invasion ability of BT-549 cells (H) transfected with si-NC and si-LINC01070 assessed by Transwell assay. p value is considered significant (**p<0.05, ***p<0.001).

In addition, clone formation assay (Figure 8A), EdU assay (Figure 8B), and Transwell assay (Figure 8C) showed that knockdown of LINC01070 inhibited breast cancer progression.

**Figure 9:**
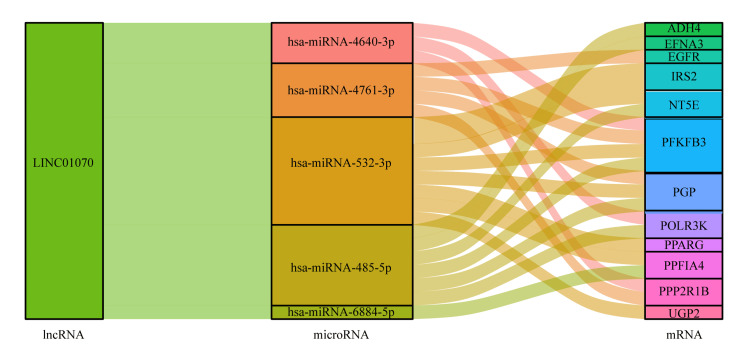
lncRNA-microRNA-mRNA prediction network.

Moreover, we constructed a lncRNA-miRNA-mRNA network graph using the Starbase database and Spearman correlation analysis to explore the possible downstream molecular mechanisms of prognosis-related GRLs (Figure 9).

## Discussion

In the current study, we first screened 41 differential GRLs from the TCGA-Breast Cancer database and the GSEA database. Nine prognostically relevant GRLs (TRHDE.AS1, LINC01070, KIF26B.AS1, AP001922.3, ALDH1L1.AS2, AL357093.2, AC134312.5, AC120498.10, AC002546.1) were identified using lasso regression analysis, and an associated prognostic risk signature was constructed and patients were divided into high-risk and low-risk groups based on expression level. Kaplan-Meier survival curves showed that patients in the high-risk group had a worse prognosis than those in the low-risk group. In addition, multivariate Cox regression analysis showed that the risk model was an independent risk factor for OS and that the risk model-based nomogram had better prognostic power. Moreover, three additional GRLs (AC134312.5, LINC01070, and KIF26B.AS1) associated with OS were subsequently identified, functional analyses showed that knockdown of LINC01070 inhibited breast cancer progression, and a network graph of LINC01070-miRNA-GRGs was constructed.

In recent years, the energy metabolism of tumor cells has received increasing attention from domestic and international researchers. Compared with normal cells, reprogramming of capacity metabolism is one of the important features of tumor cells [16]. The metabolic changes in tumors are intricate and complex, involving glucose consumption, amino acid uptake and consumption, and glycolysis/tricarboxylic acid (TCA) cycle intermediates [17, 18]. Recent studies have revealed that aerobic glycolysis plays a key role in breast cancer development and progression [19]. As an important glycolytic rate-limiting enzyme, pyruvate dehydrogenase kinase 1 (PDK1) is associated with poor prognosis and tumor cell metastasis and proliferation [20, 21]. Hexokinase (HK) is a key enzyme in the glycolytic pathway, and Brown et al. [22] found a 79% positive rate of HK2 in primary breast cancer tissues; HIF-1a binds HK2, which in turn promotes the proliferation and progression of breast cancer development by increasing glycolysis [23]. Brown et al. [24] showed that downregulation of GLUT4 decreased basal glucose uptake by cells and induced a significant increase in the proliferation of breast cancer MCF7 and MDA-MB-231 cell metabolic reprogramming, which ultimately inhibited breast cancer cell proliferation.

A growing number of studies have confirmed that lncRNAs play an important role in tumor development and can affect genes related to glucose metabolism [25, 26]. More importantly, it has been reported that many lncRNAs can regulate tumor progression by participating in the regulation of tumor glucose metabolism. LncRNA ceruloplasmin (NRCP) acts as an intermediate binding site for STAT1 and RNA polymerase II, which can lead to increased expression of downstream glucose-6-phosphate isomerase and promote glucose metabolism in tumor cells [27]. Another study found that lncRNA UCA1 can promote glycolysis by upregulating HK2 via the mTOR-STAT3/microRNA143 pathway in bladder cancer cells [28]. In breast cancer, Huang et al. [29] found that lncRNA SNHG5 can regulate BACH1 by targeting miR-299, which in turn promotes cellular glycolysis and proliferation capacity; Wang et al. [30] demonstrated that LINC01605 can promote aerobic glycolysis via LDHA in triple-negative breast cancer. Collectively, these findings suggest that lncRNAs have an important regulatory role in tumor cell glycolysis, including breast cancer cells.

Our findings provide a basis for investigating the mechanisms of glycolysis and related lncRNAs in breast cancer, and nomograms based on differential GRLs can help predict the survival probability of breast cancer patients. However, our study also has some limitations. First, our data were downloaded from the TCGA public database, the ethnicity is mainly American, and further validation in other races with a large sample is needed. Second, further in-depth molecular mechanism studies as well as in vitro and in vivo experiments are needed to validate the results of data mining.

## Conclusions

In conclusion, we identified nine lncRANs associated with glycolysis and constructed a prognostic risk model with high accuracy, and functional analyses showed that knockdown of LINC01070 inhibited breast cancer progression. Our results may provide potential targets for the treatment of breast cancer.

## References

[1] Mayayo-Peralta I, Prekovic S, Zwart W (2021). Estrogen receptor on the move: cistromic plasticity and its implications in breast cancer. Mol Aspects Med.

[2] Sung H, Ferlay J, Siegel RL, Laversanne M, Soerjomataram I, Jemal A, Bray F (2021). Global Cancer Statistics 2020: GLOBOCAN estimates of incidence and mortality worldwide for 36 cancers in 185 countries. CA Cancer J Clin.

[3] Bertucci F, Ng CK, Patsouris A (2019). Genomic characterization of metastatic breast cancers. Nature.

[4] Zubair M, Wang S, Ali N (2020). Advanced approaches to breast cancer classification and diagnosis. Front Pharmacol.

[5] Gaynor N, Crown J, Collins DM (2022). Immune checkpoint inhibitors: Key trials and an emerging role in breast cancer. Semin Cancer Biol.

[6] Tsai MC, Manor O, Wan Y (2010). Long noncoding RNA as modular scaffold of histone modification complexes. Science.

[7] Ransohoff JD, Wei Y, Khavari PA (2018). The functions and unique features of long intergenic non-coding RNA. Nat Rev Mol Cell Biol.

[8] Evans JR, Feng FY, Chinnaiyan AM (2016). The bright side of dark matter: lncRNAs in cancer. J Clin Invest.

[9] Kopp F, Mendell JT (2018). Functional classification and experimental dissection of long noncoding RNAs. Cell.

[10] Amelio I, Bernassola F, Candi E (2021). Emerging roles of long non-coding RNAs in breast cancer biology and management. Semin Cancer Biol.

[11] Pang JL, Huang FH, Zhang YH, Wu Y, Ge XM, Li S, Li X (2021). Sodium cantharidate induces apoptosis in breast cancer cells by regulating energy metabolism via the protein phosphatase 5-p53 axis. Toxicol Appl Pharmacol.

[12] Liberti MV, Locasale JW (2016). The Warburg Effect: how does it benefit cancer cells?. Trends Biochem Sci.

[13] Qin C, Lu R, Yuan M (2021). Circular RNA 0006349 augments glycolysis and malignance of non-small cell lung cancer cells through the microRNA-98/MKP1 axis. Front Cell Dev Biol.

[14] Cui Z, Sun G, Bhandari R (2021). Comprehensive analysis of glycolysis-related genes for prognosis, immune features, and candidate drug development in colon cancer. Front Cell Dev Biol.

[15] Ganapathy-Kanniappan S, Geschwind JF (2013). Tumor glycolysis as a target for cancer therapy: progress and prospects. Mol Cancer.

[16] Hanahan D, Weinberg RA (2011). Hallmarks of cancer: the next generation. Cell.

[17] Seyfried TN, Shelton LM (2010). Cancer as a metabolic disease. Nutr Metab (Lond).

[18] DeBerardinis RJ, Chandel NS (2016). Fundamentals of cancer metabolism. Sci Adv.

[19] Wu Z, Wu J, Zhao Q, Fu S, Jin J (2020). Emerging roles of aerobic glycolysis in breast cancer. Clin Transl Oncol.

[20] Velpula KK, Bhasin A, Asuthkar S, Tsung AJ (2013). Combined targeting of PDK1 and EGFR triggers regression of glioblastoma by reversing the Warburg effect. Cancer Res.

[21] Dupuy F, Tabariès S, Andrzejewski S (2015). PDK1-dependent metabolic reprogramming dictates metastatic potential in breast cancer. Cell Metab.

[22] Brown RS, Goodman TM, Zasadny KR (2002). Expression of hexokinase II and Glut-1 in untreated human breast cancer. Nucl Med Biol.

[23] Cao L, Wang M, Dong Y (2020). Circular RNA circRNF20 promotes breast cancer tumorigenesis and Warburg effect through miR-487a/HIF-1α/HK2. Cell Death Dis.

[24] Garrido P, Osorio FG, Morán J, Cabello E, Alonso A, Freije JM, González C (2015). Loss of GLUT4 induces metabolic reprogramming and impairs viability of breast cancer cells. J Cell Physiol.

[25] Xiao ZD, Zhuang L, Gan B (2016). Long non-coding RNAs in cancer metabolism. Bioessays.

[26] Liu H, Luo J, Luan S, He C, Li Z (2019). Long non-coding RNAs involved in cancer metabolic reprogramming. Cell Mol Life Sci.

[27] Rupaimoole R, Lee J, Haemmerle M (2015). Long noncoding RNA ceruloplasmin promotes cancer growth by altering glycolysis. Cell Rep.

[28] Li Z, Li X, Wu S, Xue M, Chen W (2014). Long non-coding RNA UCA1 promotes glycolysis by upregulating hexokinase 2 through the mTOR-STAT3/microRNA143 pathway. Cancer Sci.

[29] Huang SL, Huang ZC, Zhang CJ, Xie J, Lei SS, Wu YQ, Fan PZ (2022). LncRNA SNHG5 promotes the glycolysis and proliferation of breast cancer cell through regulating BACH1 via targeting miR-299. Breast Cancer.

[30] Wang W, He X, Wang Y (2022). LINC01605 promotes aerobic glycolysis through lactate dehydrogenase A in triple-negative breast cancer. Cancer Sci.

